# Associations of end-of-life preferences and trust in institutions with public support for assisted suicide evidence from nationally representative survey data of older adults in Switzerland

**DOI:** 10.1371/journal.pone.0232109

**Published:** 2020-04-23

**Authors:** Sarah Vilpert, Carmen Borrat-Besson, Gian Domenico Borasio, Jürgen Maurer

**Affiliations:** 1 Faculty of Biology and Medicine, University of Lausanne, Lausanne, Switzerland; 2 Swiss Centre of Expertise in the Social Sciences (FORS), University of Lausanne, Lausanne, Switzerland; 3 Palliative and Supportive Care Service, University of Lausanne Medical Center, Lausanne, Switzerland; 4 Faculty of Business and Economics, University of Lausanne, Lausanne, Switzerland; Universidad de la Republica, Facultad de Ciencias Sociales, URUGUAY

## Abstract

The legality of euthanasia and assisted suicide (AS) and nature of regulations of these practices remain controversial and the subject of lively debate among experts and the general public. Our study investigates attitudes and behaviours towards AS among older adults in Switzerland where the practice of AS has a relatively long history and remains rather unregulated. We aim to explore how individuals’ preferences regarding their end of life, as well as individuals’ trust in institutions involved in the practice or control of AS are associated with attitudes and behaviours towards AS. We analyse nationally representative data of adults aged 55 and over from wave 6 (2015) of the Survey of Health, Ageing and Retirement in Europe (SHARE) in Switzerland (n = 2,145). While large majorities supported current legal arrangements around AS in Switzerland (81.7%) and stated that they could consider AS for themselves under certain circumstances (61.0%), only a minority either was a member of a right-to-die organisation already (4.9%) or stated they were likely to become a member of such an organisation (28.2%). Stated preferences for control over the end of life and for maintaining essential capabilities at the end of life showed a positive association with AS-related attitudes and behaviours, whereas preferences for feeling socially and spiritually connected, as well as for not being a burden displayed a negative association with our outcomes. Higher levels of trust in one’s relative were positively associated with both support for the legality of AS and potential use of AS. A positive association was also found between trust in the Swiss legal system and support for the legality of AS. By contrast, trust in religious institutions displayed a negative association with all five AS-related attitudes and behaviours. Similarly, trust in healthcare insurance companies was negatively associated with potential use of AS. Taken together, older adults were generally supportive towards current practices regarding AS. This approval appears to be closely related to individuals’ preferences and, at different extends, to trust in social and public institutions with regard to end-of-life issues, which is relatively high in Switzerland.

## Introduction

Both the legality of euthanasia and assisted suicide (AS) as well as the adequacy of corresponding regulations to prevent abuse of these practices remain controversial and are the subject of considerable debate in medicine [1, 2], ethics [3, 4], law [5], at the intersection between medicine, humanities and social sciences [6, 7], as well as in the mainstream media [8–12]. This long-standing debate aims to balance considerations of self-determination and a “right to die”, protection of patients and healthcare providers, and other ethical, legal and medical issues that arise when ending human life.

Key arguments for euthanasia, AS and an individual’s “right to die” are often based on the moral principle of respect for patient autonomy. Patient autonomy aims to encourage and enable patients to make their own informed medical choices, including the potential ending of their lives [13]. Over the past decades, advances in medical care have resulted in substantial increases in life expectancy [14]. At the same time, medical progress has also led to an increased medicalization of health, especially at the end of life [15], which may not always be beneficial to patients [16]. Whereas some people are willing to undergo all potential treatments to sustain or extend their life for as long as possible [17], others consider certain health states as worse than death [18], and may, therefore, contemplate euthanasia or AS [19]. Yet, in most Western societies, patients’ rights of self-determination are frequently limited to the withholding or withdrawing of life-sustaining treatments.

Opposition to legalised euthanasia and/or AS is based on a number of common arguments. These concern the absolute value of human life [20], potential clinical problems and complications [21], the incompatibility of this practice with the medical deontology [22], and potential emotional distress among those involved in these practices [23], notably physicians [24]. Fears also include the so-called “slippery slope hypothesis”, i.e., a routinization of the use of euthanasia and/or AS [25], undue pressures on severely ill or elderly patients, or even the use of euthanasia against the will of the affected person, either deliberately or by error [26, 27]. In fact, opponents of euthanasia and/or AS often fear that people may be pushed to demand euthanasia or AS because family members and/or society could make them feel like a burden [26, 28] and such pressures may be stronger for more vulnerable groups such as older persons [27, 29].

Views on euthanasia also differ widely in the general population for the aforementioned reasons [29, 30]. Individuals’ attitudes toward autonomy, self-determination and control are, thereby, usually correlated with approval of euthanasia and AS. A study based on international survey data on euthanasia attitudes revealed that a higher valuation of personal autonomy increases individuals’ permissiveness towards euthanasia [28]. In addition, fears of disease progression and associated loss of autonomy and dignity, an inability to enjoy life, persistent physical or psychological suffering, and a desire for remaining in control have been shown to be key motivating factors of patients for considering or requesting euthanasia and/or AS [27, 31–33]. These factors are also associated with support for the legality of euthanasia and/or AS in the general population [27, 34]. On the other hand, fears of a "slippery slope" and worries about abusive practice of euthanasia or AS may be lower in individuals who generally trust the people and institutions involved in the practice of euthanasia or AS. Cohen et al., for example, observed a decrease in acceptance of euthanasia among Eastern European countries between 1999 and 2008 [30]. One explanation for this decrease may be a decline of trust in the state and healthcare organizations related to the socio-economical and political changes in these countries during that period [30]. Individuals have confidence in the actions of their government and other representatives of public institutions if they believe that these public institutions act in the public interest and protect people [35]. Support for the practice of euthanasia and/or AS may, therefore, be higher if individuals do not suspect any potential misuse of euthanasia and/or AS but trust the people and institutions involved in these practices.

Our study uses nationally representative survey data to assess older adults’ support for the legality of the practice of AS in Switzerland as well as their openness to consider AS for themselves under certain circumstances and their actual and planned membership in a right-to-die organisation. We further examine the association of these AS-related attitudes and behaviours with older adults’ end-of-life (EOL) preferences and levels of trust with regard to EOL issues in key social and public institutions involved in the practice or control of AS in Switzerland.

Our study makes several contributions to the literature. While previous studies have explored attitudes towards euthanasia and AS in the general population [30, 36–41], there are—to the best of our knowledge—no previous studies that have investigated both attitudes and behaviours (actual and planned membership in a right-to-die organisation) in the same population in parallel. This strategy allows to assess the associations of EOL preferences and trust in social or public institutions not just with AS-related attitudes but also corresponding behaviours. Switzerland is a particularly interesting case study for our investigation on the association between EOL preferences and trust in social or public institutions on the one hand and AS-related attitudes and behaviours on the other. Besides having a long tradition in (semi-)direct democracy that gives the public considerable power in political decision-making [42], Switzerland has a relatively long history of rather unregulated AS practice. Exploring the associations of EOL preferences and trust in institutions jointly with attitudes and behaviours towards AS gives some new insights into which groups of individuals as defined by their EOL preferences and trust in institutions are particularly supportive or sceptical regarding the practice euthanasia and AS. This may also offer scope for identifying intervention points for increasing support of the current legal arrangements in the older population in Switzerland.

### Background

Euthanasia is defined as the intentional killing of a competent person upon her voluntary request, usually performed by a physician [43]. In contrast, AS is the intentional helping of a competent person to commit suicide by providing lethal drugs for self-administration [43]. At the time of writing, euthanasia and/or AS are legal in four European countries (the Netherlands, Belgium, Luxembourg, Switzerland), in eight US states, in Canada, in Victoria (Australia) and in Colombia, while other countries such as France, Germany, Italy, and Spain are currently discussing the potential legalization of euthanasia and/or AS [44]. Most countries in which euthanasia and/or AS are legal have strongly regulated their practice and established certain safeguards. These include requiring two concurring medical opinions for any euthanasia or AS request, having a delay between the granting and carrying out of any such request, requiring psychiatric assessments of patients, and/or making restrictions on patient’s age, diagnosis or symptom state [26, 29].

In Switzerland, the practice of AS remains relatively unregulated to date, with few formal restrictions or safeguards in place to restrict the use of AS [45]. While euthanasia remains illegal (Art. 114 of the Swiss Criminal Code), the practice of AS is mainly regulated through article 115 of the Swiss Penal Code. Article 115 stipulates that assisting a person in committing suicide is not punished as long as this practice is (a) not performed for selfish reasons (e.g., obtaining an inheritance or making money) and (b) the person seeking for AS has decisional capacity. Thus, in Switzerland, the practice of AS does not require the presence of a physician. However, according to the Swiss Law of Pharmaceutical Products [46], the lethal drug (sodium pentobarbital) usually used for causing death must be provided by a physician who ought to examine the wish to die and the decisional capacity of the person seeking AS. In addition to being subject to these two laws regulating the practice of AS in Switzerland, the large majority of physicians in Switzerland (91%) are affiliated with the Swiss Medical Association (FMH), and thus bound to observe its code of ethics. The code of ethics of the FMH includes the medico-ethical guidelines of the Swiss Academy of Medical Sciences (SAMS), in particular those on the management of dying and death. The latter guidelines stipulate that AS is not considered as part of a doctor’s duties. However, physicians who wish to engage in this practice are legally authorized to do so under specific requirements mainly related to the patient’s mental capacity, persistent and not pressured wish to die, and the intractability of her/his disease or impairment [47]. The former version of the SAMS guidelines also stipulated that the patients must be “close to death” in order to receive AS. In the new SAMS guidelines of 2018, this has been replaced by the notion of “intolerable suffering”. This notion has been rejected as “too vague” by the FMH, which for the first time has refused to adopt the SAMS guidelines in its code of ethics, thus creating an “ethical uncertainty” of sorts for Swiss physicians on this matter.

Since the 1980s, not-for-profit right-to-die organisations, which advocate the right to self-determination in death, have been assisting persons with suicide under the regulation of article 115. Anyone can become a fee-paying member of a right-to-die organisation at any time, even without any immediate or future intention to request AS. Membership in such organisations grants access to their services provided that the person seeking AS meets the internal criteria of the respective right-to-die organisation [46]. Almost all AS in Switzerland are organised by one of these right-to-die organisations [48]. In 2016, AS represented 1.4% of all deaths in Switzerland and 86% of these deaths occurred in adults aged 65 years or older [49].

## Data and methods

We developed a questionnaire about EOL preferences, knowledge, attitudes and behaviours [50] that was administered as part of the 2015 data collection round (wave 6) of the Swiss version of the Survey of Health, Ageing and Retirement in Europe (SHARE) [51]. SHARE is a longitudinal, interdisciplinary and cross-national data infrastructure that comprises individual-level information on health, socio-economic status, social and family networks and other life circumstances of older persons from 27 European countries and Israel. The Swiss SHARE study was approved by the ethics committee of the canton of Vaud in March 2014 (approval number 66/14). Respondents provided informal oral consent for their participation to the SHARE study by accepting (a) to schedule a personal interview and (b) to take part in a one-hour face-to-face interview where the voluntary and confidential nature of participation in the study was reiterated.

The EOL survey was administered as a 15-page paper-and-pencil self-completion questionnaire at the end of the regular SHARE face-to-face interview. The Swiss SHARE sample is designed to be nationally representative of community-dwelling individuals aged 50 and older and their partners and is periodically refreshed to maintain its target population. Since the last refreshment sample for SHARE Switzerland was drawn in 2011, adults aged 50–54 are underrepresented in the 2015 follow-up study, as they were only included in the SHARE sample as partners. 2,806 respondents participated in the 2015 interview round in Switzerland, 94% of whom also completed our EOL questionnaire. Retaining adults aged 55 and over, we obtained a final analytical sample of 2,549 respondents for this study.

### Measures

#### Outcome variables of interest

Our study considered four measures of attitudes and behaviours regarding AS based on the following questions as outcomes: (1) “Do you support the legality of assisted suicide as is the case in Switzerland?” (2) “Can you imagine circumstances under which you would consider asking for assisted suicide yourself?” (3) “There are associations in Switzerland, such as “Exit” or “Dignitas”, which offer assisted-suicide. Are you a member of such an association?” (4) “How likely is it for you to become member of such an association some day in the future?” The three first questions could be answered by “yes” or “no”. Permissible answers to the fourth question were “for sure” and “very likely” (grouped together), as well as “not very likely” and “certainly not” (grouped together). In addition, we built a composite measure indicating current membership (3) and intended membership (4) in a right-to-die organisation, which is coded as 1 if respondents either already are a member of a right-to-die association or consider a future membership as “sure” or “very likely”, and 0 otherwise.

#### Independent variables of interest

The key independent variables of our analyses relate to EOL preferences and trust in key social and public institutions with regard to EOL issues. EOL preferences are comprehensively measured based on a 23-item survey battery in which respondents assessed the importance of different aspects of end of life for themselves on a 4-point Likert scale ranging from “not important” to “very important” (details about the 23 items are available in S1 Appendix). The 23 items are the result of an adaptation of items drawn from a literature review about EOL preferences after discussions of the Swiss SHARE team and with an expert in palliative care. Since simultaneous analysis of the 23 items would result in statistical challenges associated with high dimensionality and near multicollinearity, we summarized this information into dimensions.

While there is no standardized scale for measuring EOL preferences based on psychometric theory, the literature nevertheless suggests several important dimensions regarding what individuals usually consider important for “a good death”. We therefore assess the number of dimensions underlying the individual EOL items based on Exploratory Structural Equation Modeling (ESEM) provided by Mplus [52]. ESEM overcomes some of the limitations of the Exploratory and Confirmatory Factor Analysis (EFA and CFA), traditionally used to generate and test measurement models, by integrating the feature of the EFA and the CFA within a Structural Equation Modeling (SEM) framework [53].

Geomin rotation (epsilon = .01), WLSMV estimator and FIML were used. Eight sequential models were fit by systematically increasing the number of factors. The fit of each model was first considered separately based on chi-square (not significant), CFI (≥.95), TLI (≥.95) and RMSEA (≤.06). Nested models were then compared and a more complex model was favored if the change in robust chi-square was significant, the CFI increased by .01 or more the TLI by .009 or more and the RSMEA decreased by .015 or more as suggested by emerging guidelines [53–55]. Factors of the retained model were then interpreted based on the items that display a loading of .32 or more. Items with higher loading were considered as contributing more to the definition of the dimension. The analysis resulted in four EOL dimensions: a physical dimension expressing the importance of maintaining essential capabilities at end of life, a control dimension showing the importance of having control over end of life, a psychological dimension indicating the importance of feeling socially and spiritually connected at end of life, and a burden dimension referring to the importance of not being a burden for family and society at end of life and of contributing to others [56]. S1 Appendix presents the loadings of the EOL preferences items on the identified dimensions. Dimensions were standardized to a mean of zero and a standard deviation of 1.

To measure trust in institutions that are commonly involved in EOL issues, survey participants were asked to rate their level of trust in (1) their relatives; (2) healthcare providers; (3) Swiss healthcare system; (4) Swiss legal system; (5) healthcare insurance companies; (6) religious authorities in Switzerland on a 4-point Likert scale ranging from “completely” to “not at all”. Answers to these items were transformed into a series of dichotomous variables where the categories “completely” and “somewhat” were combined and coded as 1, whereas the combined category of “a little” and “not at all” was coded as 0.

#### Control variables

Respondents’ sociodemographic and family characteristics used as control variables included sex, age group (55–64; 65–74; 75+), education level (ISCED1997 levels 1–2, 3–4, 5–6) [57], living with a partner in the same household (yes, no), having living children (yes, no). Controls for geographical location referred to place of residence (urban vs rural areas) and language region (German, French or Italian). Respondents’ own health was measured through self-rated health status (poor/fair vs good/very good/excellent) and self-reported limitations in activities of daily living (ADLs) (no limitation vs 1+ limitation(s)). Self-reported practice of prayer distinguished individuals who never prayed from those who pray at least occasionally. Finally, experience as a healthcare proxy was measured as having at least once participated in medical decisions for a relative or a friend who was incapable of making decisions.

#### Missing values

Across the 66 variables used in our analysis, proportions of missing values ranged between 0% and 17%, with 66% of observations representing complete cases with no missing item (Table 1 details the distribution of missing values). Since the results of Little tests [58] rejected the hypothesis that our missing values were missing completely at random (MCAR), we conducted our analyses on imputed data. Specifically, multiple imputations by chained equations were used to handle the presence of missing values in our data. 200 imputed datasets were computed in order to calculate asymptotically correct margins. The imputation equations were based on all sociodemographic and family characteristics, as well as on linguistic region variables included in our study. We run logistic regressions using observations with no missing data on the respective dependent variable, which results in small variability in the outcome-specific sample sizes [59]. Parameter estimates and standard errors of logistic regression models based on imputed data sets using Rubin’s formulas [60] were compared with the results of the same regression models using complete cases (available in S2 Appendix). Resulting estimates of the different logistic regression models based on the imputed data were comparable to the corresponding estimates based on a complete case analysis, but statistical precision was higher when using imputed data.

**Table 1:** Main characteristics of the analytical sample, adults aged 55+ in Switzerland, SHARE 2015.

	Proportion %^a^	(95%-CI) ^a^	Proportion of missing responses
**Outcome variables**			
Support the legality of assisted suicide as is the case in Switzerland	81.7	(79.7,83.7)	6.4
Could consider asking for assisted suicide under certain circumstances	61.0	(58.5,63.4)	6.2
Is a member or likely to become a member of a right-to-die organisation	28.2	(26.0,30.4)	7.4
Is a member of a right-to-die organisation	4.9	(3.9,5.9)	4.2
Is likely to become a member of a right-to-die organisation^b^	23.4	(21.4,25.4)	7.8^c^
**Independent variables**			
*Standardized factors based on end-of-life (EOL) preferences*^d^			
Importance of maintaining essential capabilities at end of life	NA	NA	NA
Importance of having control over end of life	NA	NA	NA
Importance of feeling socially and spiritually connected at end of life	NA	NA	NA
Importance of not being a burden at end of life	NA	NA	NA
*Completely/somewhat trust* …			
…relatives	97.0	(96.1,97.9)	6.4
…healthcare providers	92.4	(91.0,93.7)	11.2
…Swiss healthcare system	82.2	(80.3,84.2)	14.8
…Swiss legal system	72.0	(69.6,74.4)	16.2
…healthcare insurance companies	60.5	(57.9,63.0)	17.1
…religious authorities	48.0	(45.4,50.6)	16.8
**Control variables**			
Women	50.1	(48.0,52.1)	0
*Age groups*			0
55–64	48.9	(46.3,51.5)	
65–74	28.5	(26.4,30.6)	
75+	22.6	(20.6,24.5)	
*Education level*			1.4
Low education	12.9	(11.4,14.5)	
Medium education	69.5	(67.3,71.7)	
High education	17.6	(15.7,19.5)	
Partner living in household	72.0	(69.7,74.4)	0.4
Having children	82.5	(80.5,84.6)	0.1
Urban area	47.2	(44.5,49.9)	1.3
*Linguistic region*			0
German-speaking	74.8	(72.5,77.1)	
French-speaking	22.6	(20.3,24.8)	
Italian-speaking	2.6	(1.9,3.4)	
*Health status*			
Self-rated health: (Very) good/Excellent	82.5	(80.6,84.3)	0.1
1+ limitations in activities of daily living	6.3	(5.1,7.4)	0
Practice of prayer	67.7	(65.3,70.1)	6.2
Participation in making medical decisions for relative/friend	18.5	(16.6,20.4)	5.1
n total	2,145		2,549

^a^Imputed data, weighted proportions.

^b^This question was only asked to respondents who were not member of a right-to-die organisation at the time of the survey. Imputed data n = 2,132.

^c^134 respondents were already member of a right-to-die organisation and were therefore not asked this question. The item non-response rate for this question is calculated using a denominator of n = 2,415 (exclusion of the 134 respondents).

NA for non-applicable

^d^ The factors are standardized to a mean of zero and a standard deviation of 1

#### Statistical analysis

We first reported selected characteristics of our final analytical sample based on the imputed data. Using multivariable logistic regression models, we explored the associations of AS-related attitudes and behaviours with EOL preferences and trust in institutions with regard to EOL issues. Respondents’ sociodemographic and family characteristics, geographical location, health status, practice of prayer and experience as a healthcare proxy were included in the multivariable regression models as control variables. Average partial effects (APEs) were calculated to facilitate the interpretation of the logistic regression estimates. The estimated standard errors account for clustering at the household level to correct for potential unobserved dependencies between two observations coming from the same household. All data management and statistical analyses were conducted using Stata SE 15.0 (StataCorp LLC, College Station), apart from the ESEM generating the EOL preference factor scores, which was conducted using Mplus 8.1 [52].

## Results

Table 1 presents the weighted characteristics of our analytical sample using imputed data sets. With respect to attitudinal and behavioural outcomes of interest regarding AS, 81.7% of the respondents supported the legality of AS as is the case in Switzerland and 61% reported that they would potentially consider AS for themselves under certain circumstances. In addition, 28.2% of the respondents reported they either were a member of a right-to-die organisation or planned to become one in the future. 4.9% of the respondents were already members of such an organisation at the time of the survey. Almost all respondents reported trusting their relatives (97%) with regard to EOL issues, while reported levels of trust in healthcare providers (92.4%), in the healthcare system (82.2%), as well as in the Swiss legal system (72%) were lower. Significantly fewer respondents reported trusting healthcare insurance companies (60.5%) and religious authorities (48%) with regard to EOL issues.

Table 2 presents estimated APEs based on multivariable logistic regression models for AS-related attitudes and behaviours on EOL preferences and trust in other individuals and public institutions with respect to EOL issues, controlling for sociodemographic and family characteristics, geographical location, practice of prayer, health status, and experience as a healthcare proxy. All four factors summarizing respondents’ EOL preferences were strongly associated with attitudes and behaviours towards AS. Higher reported importance of maintaining essential capabilities at the end of life and of having control over one’s end of life were positively associated with support for the legality of AS (APE maintain capabilities: 8.9, p<0.1%; APE control: 4.4, p<0.1%), as well as with potential use of AS (APE maintain capabilities: 11.3, p<0.1%; APE control: 7.0, p<0.1%); they were also positively associated with current (APE maintain capabilities: 2.3, p<0.1%; APE control: 4.5, p<0.1%) or potential future membership in a right-to-die organisation (APE maintain capabilities: 11.6, p<0.1%; APE control: 12.7, p<0.1%). Conversely, higher disclosed importance of feeling socially and spiritually connected was negatively associated with support for the legality of AS (APE: -7.9, p<0.1%), with potential use of AS (APE: -9.1, p<0.1%) and with current (APE: -3.0, p<0.1%) or potential future membership in a right-to-die organisation (APE: -11.3, p<0.1%). A higher stated importance of not being a burden to others at the end of life was also negatively associated with three outcomes: support for the legality of AS (APE: -4.7, p<0.1%), potential use of AS (APE: -5.6, p<0.1%) and with current or potential future membership in a right-to-die association (APE: -3.9, p<1%). However, higher reported importance of not being a burden to others at the end of life was not associated with current membership in a right-to-die organisation (APE: 0.0, p>5%).

**Table 2:** Average Partial Effects (APEs) based on logistic regressions of attitudes and behaviours towards assisted suicide on end-of-life preferences, as well as on trust in institutions regarding end-of-life issues, controlling for sociodemographic and family characteristics, geographical location, practice of prayer, health status, and experience as a healthcare proxy, adults aged 55+ in Switzerland, SHARE 2015.

	Support the legality of assisted suicide as is the case in Switzerland	Could consider asking for assisted suicide	Is a member or likely to become a member of a right-to-die organisation	Is a member of a right-to-die organisation	Is likely to become a member of a right-to-die organisation^c^
	APE/(ci95)	APE/(ci95)	APE /(ci95)	APE /(ci95)	APE /(ci95)
*End-of-life (EOL) preferences*: *importance of…*^a^					
maintaining essential capabilities	8.9***	11.3***	11.6***	2.3**	10.5***
	(6.4,11.4)	(8.2,14.4)	(8.7,14.6)	(0.7,3.9)	(7.6,13.3)
having control over EOL	4.4***	7.0***	12.7***	4.5***	10.1***
	(2.1,6.7)	(4.1,9.9)	(10.0,15.5)	(3.0,6.0)	(7.4,12.9)
feeling socially and spiritually connected	-7.9***	-9.1***	-11.3***	-3.0***	-9.4***
	(-10.5,-5.3)	(-12.2,-6.0)	(-14.1,-8.5)	(-4.5,-1.5)	(-12.1,-6.6)
not being a burden	-4.7***	-5.6***	-3.9**	0.0	-4.2***
	(-7.1,-2.2)	(-8.4,-2.8)	(-6.5,-1.3)	(-1.2,1.3)	(-6.7,-1.7)
*Completely/somewhat trust…*^b^					
…relatives	20.7**	15.8*	-0.7	1.2	-2.4
	(7.9,33.5)	(3.1,28.6)	(-12.3,10.8)	(-4.4,6.8)	(-14.1,9.2)
…healthcare providers	5.2	6.3	0.1	-0.1	-0.3
	(-2.2,12.6)	(-2.1,14.8)	(-7.4,7.7)	(-4.2,4.1)	(-7.5,7.0)
…Swiss healthcare system	3.3	0.2	1.0	-0.3	0.9
	(-2.3,8.9)	(-6.5,6.8)	(-4.9,6.8)	(-3.4,2.8)	(-4.6,6.4)
…Swiss legal system	4.8*	4.0	2.0	1.5	1.7
	(0.2,9.5)	(-1.5,9.6)	(-3.0,7.0)	(-1.1,4.2)	(-3.0,6.3)
…healthcare insurance companies	-2.7	-8.0**	-3.6	-2.1	-1.8
	(-6.6,1.2)	(-13.0,-3.0)	(-8.2,1.1)	(-4.6,0.4)	(-6.1,2.5)
…religious authorities	-9.8***	-9.8***	-7.6***	-3.6**	-5.5*
	(-13.6,-6.1)	(-14.5,-5.1)	(-12.1,-3.1)	(-5.9,-1.4)	(-9.7,-1.3)
n	2145	2145	2145	2145	2132

Average partial effects based on logistic regression models. All probabilities are multiplied by 100.

Asterisks indicate levels of significance:

***p<0.1%,

**p<1%,

*p<5%.

^a^ Factor scores were normalized with a mean of 0 and a standard deviation of 1. Interpretation of APEs: A one standard deviation increase in “importance of maintaining essential capabilities” implies a 8.9 percentage points increase in supporting the legality of assisted suicide as is the case in Switzerland.

^b^ Interpretation of APEs: Trust relatives increases the probability of supporting the legality of assisted suicide as is the case in Switzerland by 20.7 percentage points compared to not trusting.

^c^ Only respondents who were not member of a right-to-die organisation at the time of the survey answered this question.

The difference in sample sizes in the regression models is due to a difference in missing responses on the respective dependent variables.

Trust in one’s relatives with regard to EOL issues was positively associated with support for the legality of AS (APE: 20.7, p<1%) and with potential use of AS (APE: 15.3, p<5%), but not statistically significantly associated with current (APE: 1.2, p>5%) or potential future membership in a right-to-die organisation (APE: -0.7, p>5%). Trust in healthcare providers (APE: 5.2, p>5%) and in the Swiss healthcare system (APE: 3.3, p>5%) with regard to EOL issues were both positively, but not statistically significantly, associated with support for the legality of AS, and barely associated with current (APE healthcare providers: -0.1, p>5%; APE healthcare system: -0.3, p>5%) or potential future membership in a right-to-die organisation (APE healthcare providers: 0.1, p>5%; APE healthcare system: 1.0, p>5%). In addition, trust in healthcare providers (APE: 6.3, p>5%) was positively, but not statistically significantly, associated with potential use of AS. Furthermore, trust in the Swiss legal system was positively associated with support for the legality of AS (APE: 4.8, p<5%). It was also positively associated with potential use of AS (APE: 4.0, p>5%), and with current (APE: 1.5, p>5%) or potential future membership in a right-to-die organisation (APE: 2.0, p>5%), however in these cases the associations were not statistically significant. Trust in healthcare insurance companies, in turn, was negatively associated with all five outcomes, but only the association with potential use of AS was statistically significant (APE: -8.0, p<1%). Finally, having confidence in religious authorities was negatively and statistically significantly associated with all five favourable attitudes and behaviours towards AS.

## Discussion

Our study provides new data on older adults’ attitudes and behaviours towards AS in a context of longstanding non-physician AS practice with relatively few related regulations. One of the strengths of this study is that it not only focuses on public opinion about AS, but also investigates potential use of this EOL option and existing membership in a right-to-die organization. In addition, our study offers novel insights into the association of EOL preferences and trust in social and public institutions with regard to EOL issues with AS-related attitudes and behaviours in the older population in Switzerland. Preferences for having control over one’s end of life and maintaining essential capabilities at the end of life were strongly associated with positive attitudes and behaviours towards AS. Individuals reporting high importance of feeling socially and spiritually connected, and of avoiding to be a burden were less supportive of AS practice, and thus less likely to consider it for themselves. On the other hand, trust in social and public institutions directly and indirectly involved in the regulation of AS in Switzerland tended to be associated with more favourable attitudes towards AS, with the exception of trust in religious authorities, which was strongly negatively associated with AS-related attitudes and behaviours.

### End-of-life preferences may shape attitudes towards assisted suicide

In our study, preferences for controlling one’s end of life, e.g., attaching importance to planning the events following one’s death, to choosing where to die, to preparing one’s family for one’s death (full details is provided in S1 Appendix), were positively associated with support for the legality of AS and with potential use of AS. Similar positive associations with AS-related attitudes and behaviours were also found among respondents who considered it important to be able to maintain essential capabilities at the end of life, such as personal hygiene, self-feeding, living without pain and retaining full mental awareness. Our results are consistent with previous studies that found higher rates of acceptance of euthanasia and/or AS in people that place higher value on self-determination and physical and mental independence at the end of life, as well as in people stating stronger preferences for dying with dignity and without suffering [27, 34]. In addition, our data show that higher levels of importance attached to feeling socially and spiritually connected at the end of life, such as being able to talk about one’s fears, to be spiritually or religiously accompanied or to spend time with family and friends, were negatively correlated with support for the legality of AS and potential use of AS.

Rodríguez-Prat et al. provide a useful explanatory model for interpreting the opposing associations of different EOL preferences with support for AS [61], identifying two types of dignity related to EOL situations. Dignity based on social and spiritual values such as having a positive impact on family ties or having spiritual or religious beliefs is called “intrinsic dignity”, and contrasts with dignity based on values such as autonomy, control and quality of life, collectively referred to here as "extrinsic dignity". When confronted with serious illness and its consequences of mental and physical decline, individuals with intrinsic dignity may be more likely to maintain a positive view of themselves than those with extrinsic dignity based on autonomy and control. We can consider that respondents who placed high importance on feeling socially and spiritually connected at the end of life are more likely to have intrinsic dignity, while those who placed high importance on maintaining essential capabilities at the end of life and having control over one’s end of life are more likely to have extrinsic dignity. Since it allows control over EOL timing and escape from some physical deterioration, AS is an EOL option that may better meet the EOL-related concerns of people with extrinsic dignity.

### Fear of being a burden is not a motivation for favourable attitudes to assisted suicide

One of the arguments for the slippery slope is the fear that people will be directly or indirectly pushed to use euthanasia or AS for reasons such as not wanting to be a burden on others [26]. As the social and fiscal challenges of population aging have become a major societal concern, older adults may increasingly feel guilty of potentially becoming a burden for their relatives and society and thus may request AS for social and economic reasons. Two studies reported a positive association between approval of euthanasia in terminally-ill patients requesting it on the one hand and individual concerns of becoming a burden on the family on the other [34, 62]. In 2018, the Swiss population considered the financial sustainability of social security pensions (old-age and surviving dependents insurance, AHV) as well as of health and health insurance systems as the two most worrying current social issues [63] with population aging being a main driver of these challenges of financial sustainability. In this context, one may have expected that respondents who are concerned about being a burden should be more likely to support and consider AS for themselves, perhaps seeing it as a “duty to die” [64]. However, our data show that the self-rated importance of not being a burden does not appear to be the main motivation for supporting AS. Several factors may explain the negative associations between importance of not becoming a burden and AS-related attitudes. First, people who attach high importance to not being a burden may not support the current AS law if they fear to be pressured into AS. Second, many different situations that can be considered as burdening at the end of life and different notions of burden may impact attitudes toward AS differently. For example, individuals may not want to cause a psychological burden to their loved ones by choosing to die by AS, as witnessing an AS by a loved one can have a negative effect on the mental health of remaining family members and friends [23].

### Preference for control over end of life and fear of severe decline are positively associated with membership in a right-to-die organisation

In our study, older adults with a higher preference for controlling their end of life and for maintaining essential capabilities at their end of life were more likely to report that they plan to become or already were a member of a right-to-die organisation. Similarly, a study showed that the desire to keep control over the end of life and the time of death, as well as the fear of intolerable suffering and health decline were the main motivations for registering in a right-to-die organization in Switzerland [65]. Furthermore, individuals who died by AS shared similar attitudes in Switzerland and in Oregon. Specifically, these individuals often wished to control circumstances of and place of death, feared dependency [31, 66], and were worried about loss of dignity and future loss of quality of life and of self-care ability [31]. These similarities suggest that AS appeals especially to individuals who share the same concerns regarding their end of life.

### The general public needs to be better informed about different end-of-life options

Fears of losing control, mental and physical decline, and pain at the end of life are major concerns for most people [67]. Our study showed that people who are particularly worried about these EOL issues were more likely to have favourable attitudes and behaviours towards AS. This suggests that some people regard AS as a tool for improved self-determination, i.e., as a potential solution to end unbearable suffering and to ensure better control over their end of life [32]. Alternative solutions, such as advance directives and palliative care, may also be able to address these worries; both are available in Switzerland. However, knowledge about the existence of these options for EOL planning and care is lacking in some parts of Switzerland [68, 69]. Improved knowledge about the full range of available EOL options may potentially reduce the number of AS in Switzerland. Even high quality palliative care, however, cannot prevent some consequences of illness, such as loss of control and of autonomy [27]; thus AS may still remain a preference for some patients [29, 66, 70].

### Support for assisted suicide is positively associated with trust in institutions with regard to end-of-life issues

Previous studies have found that in countries with a high level of acceptance of euthanasia, such as in Scandinavian countries, the level of trust in the state was also high [26]. The Swiss population also shows high levels of trust in its government, but also in public institutions such as the Federal Tribunal, which is the highest court in Switzerland, and the police [63]. Our study documented similarly high levels of trust in social and public institutions with respect to EOL issues. Furthermore, Köneke showed a positive association between high levels of trust in others and euthanasia acceptance in European countries [26]. Our results corroborate this trend by showing that trust in the social and public institutions directly or indirectly involved in the good use of AS is associated with increased support for AS and potential use of AS. In particular, we found that trust in relatives was strongly positively associated with supporting the legality of AS and being able to consider AS. As families often carry out or help in performing administrative work related to AS requests in Switzerland [23, 71], trust in relatives appears to be a prerequisite for supporting AS and considering it for oneself. Similarly, we observed that trust in healthcare providers and the healthcare system was positively associated, but not statistically significant, with support for AS and potential use of AS. A number of authors assumed there would be a positive association between trust in physicians or healthcare system and euthanasia acceptance [26, 28, 72], but observed in their study an absence of [73] and even negative [26, 36, 40] associations between the two variables.

One might have expected that mistrust in healthcare services and providers in Switzerland would lead to increased support for AS. Indeed, since it is not in the hands of the medical profession, AS could be seen as a way to escape from distrusted healthcare services and providers. However, our study showed that the high support for AS practice is not the result of a deficient or distrusted healthcare system. Moreover, the positive association between trust in the Swiss legal system with regard to EOL issues and AS-related attitudes (although not all statistically significant) may appear in respondents’ beliefs that the judiciary can act as a gatekeeper against potential abuses of AS. The Swiss judiciary ought to ensure that no abuse occurs in the use of AS. When AS misuse is suspected, an investigation is opened, as in the Minelli model lawsuit [74]. Thus, our study seem to suggest that trust in social and public institutions directly or indirectly involved in the good use of AS may be a relevant determinant of AS-related attitudes.

We found negative associations between AS-related attitudes and trust in healthcare insurance companies. This association may indicate that higher levels of trust in health insurance companies may result in a lower need for concern e.g. about accessibility or potential financial consequences of intensive EOL medicine in the absence of AS, though further research is needed to better understand this pattern. At the same time, our results showed a negative association of trust in religious authorities, which can be readily explained by the strong moral disapproval for the active ending of life, based on the sanctity of life principle, of most institutionalised religions (e.g. Catholicism, Buddhism, Islam, Judaism) [75]. In fact, most studies exploring the relationship between religious dimensions on the one hand and euthanasia, physician-assisted AS or AS in general on the other reported similar negative associations between religious beliefs and voluntary active ending of life [26–28, 30, 36–38, 40, 73, 76–78].

Finally, trust in social and public institutions was weakly or not associated with potential and current membership in a right-to-die organization, with the exception of trust in religious authorities, which showed a strong negative association. Hence, while trust in institutions was associated with attitudes toward AS, these did not (yet) seem to translate into concrete action, such as actual or planned membership in a right-to-die organization. Rather, potential and current membership in a right-to-die organization appears to be more closely related to individual preferences than trust in institutions.

Our study has several limitations. First, our results rely partially on the use of imputed data to replace missing values, although a complete case analysis resulted in largely similar findings and the imputation procedure carefully followed standard practice as described in the method section. Second, some of our outcome measures refer to intentions and hypothetical future scenarios such as potentially considering AS for oneself under certain circumstances and planning to become a member of a right-to die organisation in the future. Such statements need to be treated with caution in view of their largely unknown validity for predicting future behaviours. While we know that stated intentions do often, but not always, predict behaviours [79], potential discrepancies between intentions and behaviours may be more frequent in assessments of potential future behaviours in hypothetical situations that are not completely specified (“certain circumstances”). Nonetheless, some research indicates that intentions regarding the EOL behaviours may be quite reliable. First, individuals who potentially consider AS for themselves [34], and those who registered in a right-to-die organisation [80], or died of AS [31, 66, 81] all shared common attitudes such as a wish to control their end of life and a fear of infirmity and decline at the end of life. Second, individuals’ attitudes toward death and dying in the general population appear to be relatively stable over time [36]. Third, most people who died by AS appear to have made their decisions to use AS before their illness [66].

## Conclusion

Switzerland is the country with the longest practice of AS in the world, and is characterized by relatively open regulations regarding AS. This situation raises important concerns as the number of deaths by AS in Switzerland is much higher than in states such as Oregon where AS is legal and highly-regulated [82]. In 2011, the Swiss Government reaffirmed, after more than a decade of parliamentary debates, the status quo in the legislation regulating AS, arguing that an extended legislation would not help in preventing AS abuse [83]. Rather such an extension could further legitimate the practice of AS, which is not desired [46]. According to our research results, the majority of older adults appears to be satisfied with the practice of AS in Switzerland. In addition, the high levels of approval of AS in Switzerland may be at least partially due to the high degree of trust in the functioning of social and public institutions, as well as a relatively individualistic culture that attaches considerable importance to considerations of autonomy [84]. To generate even broader support for the current practice of AS in Switzerland may require special outreach efforts to persons with strong religious beliefs and those who may be worried to be pressured into AS in order to not become a burden on others. At the same time, our study also shows that support for the legality of AS does not necessarily depend on the existence of tight regulations and comprehensive safeguards around these practices, but can also be sustained by individual preferences in a context with high levels of trust in institutions related to EOL issues.

## Supporting information

S1 Appendix (DOCX)

S2 Appendix (DOCX)

S3 Appendix (DOCX)
