# Correction: Associations of end-of-life preferences and trust in institutions with public support for assisted suicide: Evidence from nationally representative survey data of older adults in Switzerland

**DOI:** 10.1371/journal.pone.0234954

**Published:** 2020-06-18

**Authors:** Sarah Vilpert, Carmen Borrat-Besson, Gian Domenico Borasio, Jürgen Maurer

A colon is missing in the article title. The correct title is: Associations of end-of-life preferences and trust in institutions with public support for assisted suicide: Evidence from nationally representative survey data of older adults in Switzerland
