# Supplementary material for: Associations of end-of-life preferences and trust in institutions with public support for assisted suicide evidence from nationally representative survey data of older adults in Switzerland
Source: PLoS One. 2020 Apr 23;15(4):e0232109. doi: 10.1371/journal.pone.0232109 (PMC7179897; doi:10.1371/journal.pone.0232109)
Supplement: S1 Appendix — (DOCX) [file pone.0232109.s001.docx]

**S1 Appendix**

**Table** Proportions of respondents who assess end-of-life preferences as (very) important, and of missing responses in end-of-life preferences, as well as loadings of end-of-life preferences items on the four end-of-life dimensions, adults aged 55+ in Switzerland, SHARE 2015

| End-of-life (EOL) preferences: importance of … | | | EOL dimensions^a^ | | | |
| --- | --- | --- | --- | --- | --- | --- |
|  | Rates (%) of (very) important | Rates of missing responses | Maintaining essential capabilities | Having control over EOL | Feeling socially and spiritually connected | Not being a burden |
| Physical contact (e.g. hold hands) | 76.8 | 4.7 | .12 | .00 | .79 | -.13 |
| Talking about my fears | 73.7 | 4.7 | -.01 | .18 | .76 | -.10 |
| Not dying alone | 62.9 | 5.5 | .02 | .25 | .65 | -.11 |
| Receiving spiritual or religious assistance | 45.9 | 4.0 | -.32 | -.02 | .59 | .23 |
| Being able to talk or communicate with others | 90.9 | 4.0 | .42 | -.10 | .53 | .06 |
| Being at peace with others | 91.5 | 3.2 | .00 | .02 | .52 | .33 |
| Spending time with family and friends | 95.2 | 2.5 | .06 | .04 | .50 | .18 |
| Being at peace with myself | 94.6 | 3.5 | .13 | .08 | .45 | .25 |
| Deciding in advance which medical treatments | 85.5 | 4.4 | .63 | .38 | -.02 | -.09 |
| Being able to feed myself | 94.8 | 3.5 | .63 | -.13 | .03 | .30 |
| Being fully mentally aware | 87.0 | 4.5 | .61 | -.09 | .02 | .16 |
| Having confidence in my treating physician | 97.5 | 2.4 | .60 | .07 | .24 | .01 |
| Keeping clean (personal hygiene) | 96.5 | 3.6 | .58 | .04 | .08 | .14 |
| Living without pain | 93.2 | 3.4 | .52 | .11 | .02 | -.01 |
| Avoid over-treatment | 84.7 | 3.6 | .37 | .27 | -.02 | .00 |
| Being able to plan the events following my death | 62.2 | 4.6 | -.05 | .69 | .03 | .27 |
| Choosing where I die | 50.2 | 3.8 | .14 | .62 | .11 | .01 |
| Feeling that my family is prepared for my death | 72.7 | 5.5 | -.04 | .45 | .28 | .19 |
| Avoiding to be a burden on society | 75.8 | 3.6 | .11 | .02 | -.03 | .64 |
| Avoiding to be a burden on my family | 88.0 | 3.5 | .18 | .06 | -.02 | .52 |
| Having my finances in order | 91.3 | 3.8 | .15 | .36 | -.02 | .50 |
| Feeling useful to others | 79.5 | 3.6 | -.03 | -.10 | .34 | .49 |

^a^ In grey, standardized factor loadings higher than .32 or lower than -.32 are highlighted.
