# Supplementary material for: Associations of end-of-life preferences and trust in institutions with public support for assisted suicide evidence from nationally representative survey data of older adults in Switzerland
Source: PLoS One. 2020 Apr 23;15(4):e0232109. doi: 10.1371/journal.pone.0232109 (PMC7179897; doi:10.1371/journal.pone.0232109)
Supplement: S2 Appendix — (DOCX) [file pone.0232109.s002.docx]

**S2 Appendix**

**Table** Complete cases analysis. Average partial effects (APEs) based on logistic regressions of attitudes and behaviours towards assisted suicide on trust in institutions with regard to end-of-life issues, as well as on end-of-life preferences, controlling for sociodemographic and family characteristics, geographical location, practice of prayer, health status, and experience as a healthcare proxy, adults aged 55+ in Switzerland, SHARE 2015 (n=1,683)

|  | Support the legality of assisted suicide as is the case in Switzerland | Could consider asking for assisted suicide | Is a member or likely to become a member of a right-to-die organisation | Is a member of a right-to-die organisation | Is likely to become a member of a right-to-die organisation ^b^ |
| --- | --- | --- | --- | --- | --- |
|  | APE/(ci95) | APE/(ci95) | APE/(ci95) | APE/(ci95) | APE/(ci95) |
| *End-of-life (EOL) preferences: importance of…* ^a^ | |  |  |  |  |
| maintaining essential capabilities | 9.4^***^ | 12.1^***^ | 12.3^***^ | 2.1^*^ | 11.8^***^ |
|  | (6.6,12.2) | (8.7,15.5) | (9.1,15.6) | (0.4,3.8) | (8.5,15.0) |
| having control over EOL | 3.6^**^ | 7.3^***^ | 12.9^***^ | 3.8^***^ | 11.1^***^ |
|  | (1.1,6.0) | (4.2,10.5) | (10.0,15.9) | (2.3,5.3) | (8.1,14.1) |
| feeling socially and spiritually connected | -7.6^***^ | -8.9^***^ | -10.8^***^ | -2.1^**^ | -9.9^***^ |
|  | (-10.5,-4.8) | (-12.3,-5.4) | (-14.0,-7.6) | (-3.6,-0.6) | (-13.1,-6.6) |
| not being a burden | -4.6^***^ | -5.2^***^ | -3.6^*^ | 0.7 | -4.5^**^ |
|  | (-7.3,-2.0) | (-8.3,-2.2) | (-6.5,-0.8) | (-0.6,2.0) | (-7.3,-1.6) |
|  |  |  |  |  |  |
| *Completely/ somewhat trust…* | |  |  |  |  |
| ...relatives | 19.3^**^ | 12.7 | -5.8 | -0.9 | -6.0 |
|  | (5.4,33.2) | (-1.4,26.9) | (-19.0,7.4) | (-8.4,6.6) | (-19.7,7.7) |
| ...healthcare providers | 6.4 | 5.9 | 0.3 | -0.7 | 0.6 |
|  | (-1.2,13.9) | (-2.9,14.7) | (-7.7,8.3) | (-5.3,3.9) | (-7.4,8.6) |
| ...Swiss healthcare system | 4.0 | -0.1 | 0.3 | -0.6 | 0.3 |
|  | (-2.1,10.0) | (-6.9,6.8) | (-5.8,6.5) | (-3.8,2.5) | (-6.0,6.5) |
| ...Swiss legal system | 5.6^*^ | 4.2 | 2.5 | 2.7 | 1.1 |
|  | (0.6,10.6) | (-1.6,10.0) | (-2.7,7.7) | (-0.0,5.3) | (-4.1,6.3) |
| ...healthcare insurances | -3.5 | -6.8^**^ | -2.6 | -1.7 | -1.4 |
|  | (-7.5,0.5) | (-12.0,-1.7) | (-7.5,2.3) | (-4.1,0.7) | (-6.3,3.5) |
| ...religious authorities | -10.5^***^ | -11.7^***^ | -8.9^***^ | -4.4^***^ | -6.1^**^ |
|  | (-14.5,-6.6) | (-16.7,-6.7) | (-13.6,-4.1) | (-6.6,-2.2) | (-10.8,-1.5) |
| *Control variables* |  |  |  |  |  |
| *Sociodemographic characteristics* | |  |  |  |  |
| Women | -0.5 | -6.2^**^ | -2.5 | 0.9 | -3.6 |
|  | (-4.1,3.0) | (-10.6,-1.7) | (-6.8,1.8) | (-1.0,2.9) | (-7.9,0.7) |
| Age groups |  |  |  |  |  |
| 55-64 (ref.) | - | - | - | - | - |
| 65-74 | 4.7^*^ | 1.6 | 5.2^*^ | 1.9 | 4.3 |
|  | (0.8,8.7) | (-3.6,6.7) | (0.5,10.0) | (-0.4,4.1) | (-0.5,9.1) |
| 75+ | -2.6 | -5.7 | -3.1 | 6.1^**^ | -7.2^*^ |
|  | (-7.7,2.5) | (-12.0,0.7) | (-8.8,2.6) | (2.3,9.9) | (-12.8,-1.5) |
| *Education level* |  |  |  |  |  |
| Low education (ref.) | - | - | - | - | - |
| Medium education | 4.1 | 5.6 | 6.8^*^ | 3.3^**^ | 4.7 |
|  | (-1.3,9.6) | (-1.5,12.7) | (0.6,13.1) | (1.0,5.5) | (-1.6,10.9) |
| High education | 10.3^**^ | 11.3^**^ | 14.3^***^ | 5.7^**^ | 10.7^**^ |
|  | (3.9,16.7) | (2.9,19.8) | (6.4,22.2) | (2.1,9.4) | (3.0,18.5) |
| Partner living in household | 0.4 | 2.4 | 1.4 | 2.1 | -0.5 |
|  | (-4.0,4.9) | (-3.3,8.2) | (-4.0,6.9) | (-0.5,4.8) | (-6.0,5.0) |
| Having children | 0.2 | -0.9 | -1.0 | -5.8^**^ | 3.2 |
|  | (-4.7,5.1) | (-6.9,5.1) | (-6.8,4.8) | (-10.0,-1.5) | (-2.2,8.6) |
| *Cultural characteristics* |  |  |  |  |  |
| Urban area | -2.1 | -3.4 | -1.3 | 0.2 | -1.5 |
|  | (-5.8,1.6) | (-7.9,1.2) | (-5.5,3.0) | (-2.1,2.5) | (-5.8,2.7) |
| Linguistic region |  |  |  |  |  |
| German-speaking (ref.) | - | - | - | - | - |
| French-speaking | -3.4 | -15.3^***^ | -3.4 | 0.7 | -3.9 |
|  | (-8.2,1.5) | (-21.6,-9.1) | (-8.8,2.0) | (-2.7,4.0) | (-9.1,1.3) |
| Italian-speaking | -15.0^*^ | -11.6 | -1.5 | -2.2 | -0.8 |
|  | (-27.6,-2.5) | (-25.3,2.1) | (-14.6,11.5) | (-8.3,3.8) | (-13.2,11.5) |
| Practice of prayer | -8.8^***^ | -11.1^***^ | -7.1^**^ | -1.9 | -6.1^*^ |
|  | (-12.5,-5.0) | (-16.0,-6.2) | (-11.9,-2.3) | (-4.4,0.5) | (-10.8,-1.3) |
| *Experiential characteristics* | |  |  |  |  |
| Self-rated health: (Very) good/Excellent | 1.7 | 6.3 | -0.0 | -0.9 | 0.3 |
|  | (-3.2,6.6) | (-0.1,12.7) | (-5.9,5.8) | (-4.1,2.2) | (-5.5,6.1) |
| 1+ limitations in the activities of daily living | -0.8 | -1.4 | 5.6 | 1.1 | 5.8 |
|  | (-8.2,6.6) | (-11.5,8.7) | (-4.2,15.4) | (-4.6,6.8) | (-4.3,15.8) |
| Participation in making medical decisions for relative/friend | 2.6 | 1.5 | 3.0 | 3.2^*^ | 1.1 |
|  | (-1.9,7.0) | (-4.1,7.0) | (-2.1,8.1) | (0.1,6.3) | (-3.9,6.1) |
| n (listewise deletion) | 1683 | 1683 | 1683 | 1683 | 1592 |

Average partial effects based on logistic regression models. All probabilities are multiplied by 100.

Asterisks indicate levels of significance: ***p<0.1%, **p<1%, *p<5%.

^a^ Dimensions of end-of-life preferences were normalized with a mean of 0 and a standard deviation of 1.

^b^ Only respondents who were not member of a right-to-die organisation at the time of the survey answered this question.
