# Supplementary material for: Associations of end-of-life preferences and trust in institutions with public support for assisted suicide evidence from nationally representative survey data of older adults in Switzerland
Source: PLoS One. 2020 Apr 23;15(4):e0232109. doi: 10.1371/journal.pone.0232109 (PMC7179897; doi:10.1371/journal.pone.0232109)
Supplement: S3 Appendix — (DOCX) [file pone.0232109.s003.docx]

**S3 Appendix**

**Table** Average partial effects (APEs) based on logistic regressions of attitudes and behaviours towards assisted suicide on trust in institutions regarding end-of-life issues, as well as on end-of-life preferences, controlling for sociodemographic and family characteristics, geographical location, practice of prayer, health status, and experience as a healthcare proxy, adults aged 55+ in Switzerland, SHARE 2015

|  | Support the legality of assisted suicide | Can imagine to ask for assisted suicide | Is a member or likely to become a member of a right-to-die organisation | Is a member of a right-to-die organisation | Is likely to become a member of a right-to-die organisation^b^ |
| --- | --- | --- | --- | --- | --- |
|  | b/ci95 | b/ci95 | b/ci95 | b/ci95 | b/ci95 |
| *End-of-life (EOL) preferences: importance of…* ^a^ | | |  |  |  |
| maintaining essential capabilities | 8.9^***^ | 11.3^***^ | 11.6^***^ | 2.3^**^ | 10.45^***^ |
|  | (6.3,11.4) | (8.2,14.4) | (8.7,14.5) | (0.7,3.9) | (7.64,13.25) |
| having control over EOL | 4.4^***^ | 7.0^***^ | 12.7^***^ | 4.5^***^ | 10.13^***^ |
|  | (2.1,6.7) | (4.1,9.9) | (10.0,15.5) | (3.0,6.0) | (7.40,12.85) |
| feeling socially and spiritually connected | -7.9^***^ | -9.1^***^ | -11.3^***^ | -3.0^***^ | -9.39^***^ |
|  | (-10.5,-5.3) | (-12.2,-6.0) | (-14.1,-8.5) | (-4.5,-1.5) | (-12.14,-6.64) |
| not being a burden | -4.7^***^ | -5.6^***^ | -3.9^**^ | 0.0 | -4.22^***^ |
|  | (-7.1,-2.2) | (-8.4,-2.8) | (-6.5,-1.3) | (-1.2,1.3) | (-6.72,-1.72) |
| *Completely/ somewhat trust…* | |  |  |  |  |
| ...relatives | 20.7^**^ | 15.8^*^ | -0.7 | 1.2 | -2.44 |
|  | (7.9,33.5) | (3.1,28.6) | (-12.3,10.8) | (-4.4,6.8) | (-14.06,9.18) |
| ...healthcare providers | 5.2 | 6.3 | 0.1 | -0.1 | -0.27 |
|  | (-2.2,12.6) | (-2.1,14.8) | (-7.4,7.7) | (-4.2,4.1) | (-7.52,6.98) |
| ...Swiss healthcare system | 3.3 | 0.2 | 1.0 | -0.3 | 0.88 |
|  | (-2.3,8.9) | (-6.5,6.8) | (-4.9,6.8) | (-3.4,2.8) | (-4.64,6.40) |
| ...Swiss legal system | 4.8^*^ | 4.0 | 2.0 | 1.5 | 1.67 |
|  | (0.2,9.5) | (-1.5,9.6) | (-3.0,7.0) | (-1.1,4.2) | (-2.99,6.34) |
| ...healthcare insurances | -2.7 | -8.0^**^ | -3.6 | -2.1 | -1.79 |
|  | (-6.6,1.2) | (-13.0,-3.0) | (-8.2,1.0) | (-4.6,0.4) | (-6.11,2.54) |
| ...religious authorities | -9.8^***^ | -9.8^***^ | -7.6^***^ | -3.6^**^ | -5.50^*^ |
|  | (-13.6,-6.1) | (-14.5,-5.1) | (-12.1,-3.1) | (-5.9,-1.4) | (-9.70,-1.30) |
|  |  |  |  |  |  |
| *Control variables* |  |  |  |  |  |
| *Sociodemographic characteristics* | |  |  |  |  |
| Women | -0.3 | -3.2 | 1.4 | 1.3 | -0.28 |
|  | (-3.4,2.8) | (-7.2,0.8) | (-2.4,5.2) | (-0.4,3.1) | (-3.96,3.40) |
| Age groups |  |  |  |  |  |
| 55-64 (ref.) | - | - | - | - | - |
| 65-74 | 4.6^*^ | 0.8 | 5.2^*^ | 2.3^*^ | 3.58 |
|  | (0.8,8.3) | (-4.0,5.6) | (0.8,9.5) | (0.2,4.4) | (-0.63,7.80) |
| 75+ | -2.3 | -4.4 | -0.8 | 7.1^***^ | -6.26^**^ |
|  | (-6.8,2.2) | (-10.0,1.2) | (-5.8,4.2) | (3.9,10.4) | (-10.94,-1.57) |
| *Education level* |  |  |  |  |  |
| Low education (ref.) | - | - | - | - | - |
| Medium education | 3.1 | 5.6 | 8.4^**^ | 4.1^***^ | 5.59^*^ |
|  | (-1.8,8.0) | (-0.6,11.8) | (3.0,13.8) | (2.2,6.0) | (0.48,10.70) |
| High education | 9.5^**^ | 13.2^***^ | 15.1^***^ | 6.2^***^ | 11.25^***^ |
|  | (3.6,15.3) | (5.6,20.7) | (8.1,22.1) | (2.8,9.5) | (4.56,17.93) |
| Partner living in household | 0.6 | 4.5 | 2.5 | 1.1 | 1.76 |
|  | (-3.3,4.6) | (-0.7,9.6) | (-2.3,7.3) | (-1.3,3.5) | (-2.81,6.33) |
| Having children | -0.7 | -0.3 | -0.9 | -4.1^*^ | 1.66 |
|  | (-5.1,3.8) | (-6.0,5.3) | (-6.2,4.4) | (-7.6,-0.6) | (-3.22,6.55) |
| *Cultural characteristics* |  |  |  |  |  |
| Urban area | -1.1 | -0.8 | 0.8 | 0.9 | -0.07 |
|  | (-4.4,2.3) | (-5.0,3.3) | (-3.1,4.7) | (-1.3,3.0) | (-3.76,3.62) |
| *Linguistic region* |  |  |  |  |  |
| German-speaking (ref.) | - | - | - | - | - |
| French-speaking | -1.9 | -14.3^***^ | -1.2 | 0.2 | -1.55 |
|  | (-6.2,2.4) | (-20.0,-8.6) | (-6.2,3.9) | (-2.7,3.2) | (-6.20,3.10) |
| Italian-speaking | -14.6^**^ | -11.9 | 0.8 | -1.5 | 0.39 |
|  | (-25.5,-3.8) | (-23.9,0.0) | (-10.7,12.3) | (-7.3,4.4) | (-10.20,10.97) |
| Practice of prayer | -8.6^***^ | -10.2^***^ | -7.1^**^ | -1.5 | -6.19^**^ |
|  | (-12.2,-4.9) | (-14.8,-5.6) | (-11.6,-2.6) | (-3.8,0.7) | (-10.54,-1.84) |
| *Experiential characteristics* | |  |  |  |  |
| Self-rated health: (Very) good/Excellent | 1.3 | 2.3 | -2.4 | -2.2 | -0.28 |
|  | (-3.0,5.6) | (-3.2,7.7) | (-7.6,2.8) | (-5.1,0.7) | (-5.23,4.66) |
| 1+ limitations in the activities of daily living | -1.7 | -1.3 | 6.9 | 1.5 | 8.54 |
|  | (-8.3,4.9) | (-9.7,7.1) | (-1.5,15.3) | (-2.6,5.6) | (-0.24,17.31) |
| Participation in making medical decisions for relative/friend | 4.2^*^ | 2.5 | 4.8^*^ | 3.3^*^ | 3.05 |
|  | (0.2,8.3) | (-2.7,7.6) | (0.0,9.6) | (0.4,6.2) | (-1.49,7.60) |
| n^c^ | 2145 | 2145 | 2145 | 2145 | 2132 |

Average partial effects based on logistic regression models. All probabilities are multiplied by 100.

Asterisks indicate levels of significance: ***p<0.1%, **p<1%, *p<5%.

^a^ Factor scores were normalized with a mean of 0 and a standard deviation of 1.

^b^ Only respondents who were not member of a right-to-die organisation at the time of the survey answered this question.

^c^ The difference in sample sizes in the regression models is due to a difference in missing data on the dependent variables.
